# [6]-Gingerol-Derived Semi-Synthetic Compound SSi6 Inhibits Tumor Growth and Metastatic Dissemination in Triple-Negative Breast Cancer Xenograft Models

**DOI:** 10.3390/cancers13122855

**Published:** 2021-06-08

**Authors:** Liany Luna-Dulcey, James Almada da Silva, Veronica Jimenez-Renard, Eduardo Caleiras, Silvana Mouron, Miguel Quintela-Fandino, Marcia R. Cominetti

**Affiliations:** 1 Laboratory of Biology of Aging (LABEN), Department of Gerontology, Federal University of São Carlos (UFSCar), CEP 13565-905 São Carlos–SP, Brazil; mcominetti@ufscar.br; 2 Department of Pharmacy, Federal University of Sergipe (UFS), CEP 49400-000, Av. Gov. Marcelo Deda, 330–São José, Lagarto–SE, Brazil; jamesalmada@hotmail.com; 3 Breast Cancer Clinical Research Unit, Spanish National Cancer Research Center (CNIO), CP 28029 Madrid, Spain; vjimenezr@cnio.es (V.J.-R.); smouron@cnio.es (S.M.); mquintela@cnio.es (M.Q.-F.); 4 Histopathology Unit, Spanish National Cancer Research Center (CNIO), CP 28029 Madrid, Spain; ecaleiras@cnio.es

**Keywords:** [6]-gingerol, acute toxicity, metastatic progression, semi-synthetic compound, triple-negative breast cancer, xenograft model

## Abstract

**Simple Summary:**

Triple-negative breast cancers (TNBC) represent approximately 15% of all breast cancers and lack the expression of a defined molecular target. This absence makes this subtype of cancer difficult to treat and control. Current chemotherapy drugs cause various side effects and toxicities that can jeopardize the quality of life of patients with TNBC cancer. Therefore, this research focuses on a new semi-synthetic compound derived from [6]-gingerol, where we demonstrate that it does not cause significant toxic effects *in vivo* and, more importantly, we demonstrate its antitumor and antimetastatic effects using preclinical xenograft models simulating two clinical scenarios of a woman with breast cancer.

**Abstract:**

Breast cancer metastasis is the most common cause of cancer death in women worldwide. Triple-negative breast cancers (TNBC) form a heterogeneous group of tumors that have higher relapse rates and poorer survival compared to other breast cancer subtypes. Thus, this work reports the antitumor and antimetastatic activities of a [6]-gingerol-derived semi-synthetic compound named SSi6 on MDA-MB-231 TNBC cells using xenograft models. SSi6 did not cause toxic effects *in vivo* as demonstrated by body weight and hematological and histological evaluations. From the orthotopic xenograft model, we demonstrated that SSi6 slows and inhibits the growth of the primary tumor, as well as prevents metastatic spontaneous progression from lymph nodes to the lungs. Moreover, a second xenograft model with resection of the primary tumor showed that SSi6 also blocks the progression of metastases from the lymph nodes to other visceral organs. Taken together, our results demonstrate that SSi6 is a promising compound to be investigated in other preclinical and clinical models to be applied as a complementary therapy for TNBC.

## 1. Introduction

At present, non-communicable diseases are responsible for most global deaths [1,2]. Among these diseases, cancer is one of the main causes of morbidity and mortality worldwide, preceded only by cardiovascular diseases [3,4]. Breast cancer, specifically triple-negative breast cancer (TNBC), is a heterogeneous subtype with limited therapeutic options and is extremely aggressive, which is correlated with poor prognosis and high mortality rates. This unfavorable condition in comparison with other subtypes is mainly due to the lack of the expression of estrogen (ER-) and progesterone (PR-) receptors, and human epidermal growth factor 2 (HER2-) [5]. Additionally, due to its high heterogeneity and molecular differences, TNBC has been associated with significant variation in the clinical outcome of the patients. Recently, studies have uncovered the role of androgen receptors (ARs) in the development, recurrence, and prognosis of TNBC [6,7,8]. Although little is known about the biological role of ARs and their mechanism of action in breast cancer, these observations have increasingly led many researchers to focus their studies on this nuclear receptor as a new therapeutic target for breast cancer patients, mainly in TNBC [9,10,11].

Metastatic breast cancer (called stage IV or advanced breast cancer) is responsible for 90% of deaths among women [12]. The metastasis in TNBC occurs preferentially to the visceral organs, including lung, liver, adrenals, and brain [13,14]. Thus, the development of antitumor drugs that inhibit the growth of the primary tumor and, consequently, block the progression of the metastasis process to vital organs is one of the greatest challenges in the area. In addition, the resistance to the treatment and the lack of therapeutic selectivity of the conventional drugs, which results in diverse collateral effects, are the main drawbacks of actual curative therapy [15,16]. In this sense, researches to discover new drugs with higher selectivity towards neoplastic cells, fewer secondary effects, and capable of inhibiting the metastasis progression are extremely relevant for breast cancer treatment.

Natural compounds or their modified variants, such as semi-synthetic products, have been widely used for the treatment of various diseases, including breast cancer [17,18]. Gingerols are a group of biologically active phenolic compounds extracted from ginger’s roots (*Zingiber officinale* Roscoe), [6]-gingerol (6G) being the most representative. This compound was modified by a reaction with 2,4-dinitrophenylhydrazone acetone (a hydrazone chemically and biologically active) [19,20], resulting in the formation of a semi-synthetic compound called SSi6. The biological activities of SSi6 on MDA-MB-231 TNBC cells *in vitro* were recently reported by our group and were related to the inhibition of migration, invasion, and induction of cell cycle arrest and cell death [21,22]. In all parameters investigated, SSi6 was more efficient than its natural analogous 6G, showing promising results to be applied in animal models. Within this context, this work correlated the results previously obtained *in vitro* with *in vivo* assays by using xenograft models. Herein, we demonstrate that SSi6 inhibits tumor growth and blocks the simultaneous progression of multi-organ metastases *in vivo*.

## 2. Materials and Methods

### 2.1. Isolation, Synthesis and Purification

SSi6-*trans* (Figure S1A) and SSi6-*cis* (Figure S1B) were isomers synthesized and purified by classic liquid chromatography, as described early, with some modifications [23]. Gingerol (400 mg) and 2,4-dinitrophenylhydrazine (280 mg) were reacted in a flask at 0 °C, using anhydrous methanol as solvent, molecular sieve 3A, and hydrochloric acid (HCl) as the catalyst, under stirring for 60 min. Products extraction was performed with dichloromethane after the addition of cold water. Compounds isolation was performed by liquid chromatography on silicon oxide (SiO_2_) column (15.0 × 3.4 cm i.d.) using n-hexane/ethyl acetate, 60:40 (*V/V*). Twenty fractions (30 mL) were collected and monitored by thin-layer chromatography (TLC). The SSi6-*trans* and SSi6-*cis* were identified in the fractions 10–16 and 19–20, respectively. The compounds structures were elucidated by 1H NMR (Figure S2A,B) using a Bruker DRX400 instrument (Bruker, Billerica, MA, USA), operating at 400 MHz for 1H spectra, with TMS as internal standard and by electrospray ionization-ion trap mass spectrometry (ESI-MS/MS, Bruker model Esquire 6000) in positive mode (Figure S3A,B).

### 2.2. Chemical

According to the calculated values of the half maximal inhibitory concentration of cell viability (IC_50_), the isomer SSi6-*trans* presented higher cytotoxicity on MDA-MB-231 TNBC cells, compared to the SSi6-*cis* (Figure S4). SSi6-*trans* (hereinafter called SSi6) was chosen for the *in vitro* and *in vivo* studies. For *in vitro* assays, a stock solution of SSi6 was prepared in 100% of Dimethyl Sulfoxide (DMSO), and then convenient aliquots were added to the proper culture medium to prepare different concentrations of SSi6. In all concentrations, the final percentage of DMSO was 0.5% (*V/V*). On the other hand, for *in vivo* experiments, the composition of SSi6 doses was 90% physiological serum + 5% DMSO + 5% Tween 80.

### 2.3. Cell Lines

Selected cell lines were provided from American Type Culture Collection (ATCC) and preserved at 37 °C in an incubator with 5% carbon dioxide (CO_2_). TNBC cell lines including MDA-MB-231, MDA-MB-468, MDA-MB-157, Hs578T, HCC1937, and CAL-120 were maintained in high glucose Dulbecco’s Modified Eagle’s Medium (DMEM) and used in the preliminary experiments, in addition to cell lines BT-549, HCC1143, and HCC38 that were maintained in Roswell Park Memorial Institute (RPMI-1640). Both mediums used were supplemented with 10% Fetal Bovine Serum (FBS; Sigma-Aldrich, Darmstadt, Germany). Human kidney HEK-293T cells (normal cells; selectivity control) were maintained in DMEM supplemented with 10% FBS. Finally, the MDA-MB-231 Luminescent cell line (MDA-MB-231/Luc) used in xenograft models was generated by stable transfection with a plasmid encoding firefly luciferase (pGL.4.51 luciferase reporter vector; Promega), as previously described [24].

### 2.4. In Vitro Assays

#### 2.4.1. Viability

The effects of SSi6 on the viability of TNBC and normal cell lines were determined by using CellTiter 96^®^ AQueous One Solution Assay (MTS; Promega, Madison, WI, USA) following the manufacturer’s instructions. Cells were seeded (5 × 10^3^ cells/100 μL) into black 96-well plates. Afterward, the cells were exposed to increasing concentrations of SSi6 (3.12–100 μM) for 72 h. After treatment, the medium was removed, and cells were treated with MTS for 15 min. Luminescence was measured using VICTOR Multilabel Plate Reader (Perkin Elmer’s, Waltham, MA, USA) at 544 nm. Viability assay was analyzed in comparison to the control cells treated with 0.5% DMSO.

#### 2.4.2. Cytotoxicity by Colonies

TNBC cell lines were seeded at a density of 3 × 10^3^/well in 12-well plates and incubated at 37 °C and 5% CO2 overnight (24 h). Then, the cells were treated with different concentrations (5, 10, 12.5, and 15 μM) of SSi6 for 10 days steadily. After treatment, cells were fixed and stained with 0.1% (*W/V*) crystal violet in 10% (*V/V*) methanol. All experiments were performed using biological and technical triplicates.

### 2.5. In Vivo Studies

#### 2.5.1. Acute Toxicity and Tolerance Assays

Animals were provided from the Spanish National Cancer Research Center (CNIO) animal house. *In vivo* experiments were approved by the CNIO Ethics Committee and performed following the International Guiding Principles for Biomedical Research Involving Animals developed by the Council for International Organizations of Medical Sciences (PROEX 387/15). Female wild-type FVB mice (susceptible to leukemia Virus B; *Mus musculus*), 6–8 weeks old and weighing approximately 19–21 g, were used to determine the acute toxicity of SSi6. Animals were kept in a climate-controlled environment (22 ± 2 °C) with cycles of 12 h light/dark and with ad libitum access to food and water. Animals were divided into four groups of five animals each (n = 5): group 1—control (vehicle 90% physiological serum + 5% DMSO + 5% Tween 80); group 2—(SSi6 5 mg/kg); group 3—(SSi6 10 mg/kg) and group 4—(SSi6 15 mg/kg). Vehicle and SSi6 doses were administered intraperitoneally (i.p., 100 µL) three times a week, totaling 4 weeks of treatment.

After administration, animals were examined individually to check possible toxicity symptoms, e.g., dehydration, rough coat, hunched posture, ataxia, bodyweight loss, pain, or death. At the end of the experiment, mice were euthanized, and organs (large and small intestine, bone/bone marrow, heart, spleen, liver, and kidneys) and blood samples were collected for histology and toxicity analysis. Thus, for histological analysis, the collected organs were kept in formalin 10% for 24 h to start the histologic process. Next, blood samples (~50 µL) collected via facial/submandibular vein puncture into EDTA-coated tubes (BD Biosciences, Vacutainer) were used to verify possible alterations in cells blood by SSi6. Next, an aliquot (~3 µL) of the blood of each animal was smeared onto a clean microscope slide, leave to air-dry, fixed with absolute methanol for 3 min, and then stained with May Grunwald Giemsa.

#### 2.5.2. Histological Analyses

The collected organs (large and small intestine, bone/bone marrow, heart, spleen, liver, and kidneys) for the toxicity analyses were kept in 10% buffered formalin for 24 h and submitted to a histological procedure comprising dehydration, clarification and impregnation stages. After this, the organs were embedded in paraffin, and cut longitudinally (5 µm) for the staining process with hematoxylin and eosin (H&E) for morphological and structural analyses. Images from each slide were captured and analyzed in the ZEISS AXIO Imager.M2 Microscope (ZEISS, Jenna, Germany).

#### 2.5.3. Orthotopic Xenograft Model—Without Primary Tumor Resection

The inhibition of tumor growth and spontaneous metastases formed was evaluated. Six weeks-old female athymic nude mice (Hsd: Athymic Nude-Foxn1nu) were purchased from Charles River Laboratories. MDA-MB-231/Luc cells (1 × 10^6^ cells) were re-suspended in 50 µL of PBS and matrigel (1:1 *V/V*) and injected at the right and left mammary tissue subcutaneously. Before euthanasia, blood samples were collected via facial/submandibular and analyzed in hematological counter LaserCell equipment (CVM-LaserCell HT5, Spain). In this model, two groups of six animals each were studied (n = 6): group 1—control (vehicle 90% physiological serum + 5% DMSO + 5% Tween 80) and group 2—(SSi6 15 mg/kg). Animals were maintained at a constant temperature (24 °C) under a 12 h light/dark cycle in individually ventilated cages equipped with special filters and controlled input of humidity, particulate matter, and gas concentration. Tumor growth was monitored three times a week by a digital caliper, and when a tumor volume was around 200 mm^3^, mice were randomly divided into corresponding groups. SSi6 was administered daily by intraperitoneal injection (i.p., 100 µL). Tumor volumes were calculated using the following Equation (1) [24]:

(1) $$V\;\left(mm^{3}\right)=\frac{\left(D-d2\right)}{2}$$

where D and d are the largest and the shortest tumor diameter measured, respectively. Moreover, growth inhibition (TGI) percent was calculated according to Equation (2):

(2) $$\mathrm{TGI}\;\left(\%\right)=\left[1-{\left(T_{F}/T_{0}\right)}_{A}/{\left(T_{F}/T_{0}\right)}_{V}\right]\;\times \;100$$

where TF is the time point analyzed, T0 is the initial time, A is the corresponding drug/compound, and V is the vehicle [25]. *In vivo* bioluminescence analyses (Xenogen IVIS-200 System, Alameda, CA, USA) were performed once a week to analyze metastases formed in axillary lymph nodes and the thorax area (see Section 2.5.5). Finally, mice were euthanized in a CO_2_ chamber when tumor volume reached the humane endpoint (1500 mm^3^).

#### 2.5.4. Metastasis Xenograft Model—Primary Tumor Resection

Similar to the last model, MDA-MB-231/Luc cells (1 × 10^6^ cells) were re-suspended in 50 µL of PBS and matrigel (1:1 *V/V*), injected at the right and left mammary tissue subcutaneously. When tumors volume reached ~500 mm^3^, mice were anesthetized with 2–4% inhaled isoflurane in oxygen (O_2_), and tumors were resected using an aseptic technique with closure using interrupted 7.0 polypropylene sutures. After tumor resection, animals were separated in two groups (vehicle n = 11 and SSi6 15 mg/kg n = 15). All animals received analgesia with buprenorphine 0.1 mg/kg subcutaneously every 8 h for 3 days. After 1 week of recuperation, the treatment was initiated daily by intraperitoneal injections (SSi6 15 mg/kg, i.p., 100 µL). Bodyweight was monitored three times a week before and after primary tumor resection. *In vivo* bioluminescence was performed once a week to analyze metastases formed in the upper segment (lymph nodes and thorax) and lower segment (abdominal area/primary tumor regrowth). Mice were euthanized in a CO_2_ chamber as soon as they showed signs of distress and *ex vivo* of organs analysis (lung and heart, spleen, liver, and kidneys) was performed by bioluminescence and H&E stains (see bioluminescence imaging of *ex vivo* organs and histological analyzes).

#### 2.5.5. *In Vivo* Bioluminescence Imaging

For bioluminescence imaging (measured by using the IVIS system 200 series), 200 µL of D-luciferin (15 mg/mL; BioThema BT11-1000K) was injected intraperitoneally, and mice were anesthetized with 2–4% inhaled isoflurane in O_2_. To visualize axillary lymph nodes and thorax metastases in the orthotopic xenograft model, the primary tumor was shielded with a black tissue to block the tumor’s high luminescence that would prevent the lower luminescence detection of the metastatic cell [26]. Living Image software 4.7.3 was used to compute regions of interest (ROI) and integrate the total bioluminescence signal in each ROI. Data were analyzed using total flux (photons/second) in the ROIs, and this value was scaled to a comparable background value (from a luciferin-injected mouse with no tumor cells).

#### 2.5.6. Bioluminescence Imaging of *Ex Vivo* Organs

Before euthanasia, 200 µL of D-Luciferin (15 mg/mL) was injected intraperitoneally. After 5 min of distribution of substrate, the mice were euthanized in a CO_2_ chamber, and then spleen, liver, kidneys, and lungs were quickly removed and analyzed by using IVIS Spectrum system 200 series (Xenogen, Alameda, CA, USA).

#### 2.5.7. Histopathology

To confirm the presence of tumor cells, various organs (spleen, liver, kidneys, lung and heart, axillary lymph nodes) were removed during necropsy and stored in 10% formalin solution, immediately after *ex vivo* bioluminescence imaging. These organs were embedded in paraffin, sectioned and stained with H&E to be analyzed by microscopy using a ZEISS AXIO Imager.M2 (ZEISS, Jenna, Germany ). Additionally, axillary lymph nodes were also analyzed by immunohistochemistry (IHC) using an anti-human pan-cytokeratin (CK) clone AE1/AE3 (Dako products, IR053).

### 2.6. Statistics

Each *in vitro* experiment was performed independently in biological and technical triplicates to guarantee the reliability and reproducibility of the results. Data were analyzed by one-way ANOVA by using the GraphPad Prism^®^ Software 7.0 (Intuitive Software for Science, Sunnyvale, CA, USA). For *in vivo* survival graph, groups were calculated by Kaplan–Meier survival curve comparisons, as well as the *p* values derived from the log-rank test. Experiments of xenograft models were compared using a Student’s *t*-test using GraphPad Prism^®^ Software 7.0 (San Diego, CA, USA). All data are presented as the mean ± standard deviation (SD).

## 3. Results

### 3.1. Effect of SSi6 on Cytotoxicity and Viability in TNBC Cell Lines: In Vitro Assays

**Figure 1:**
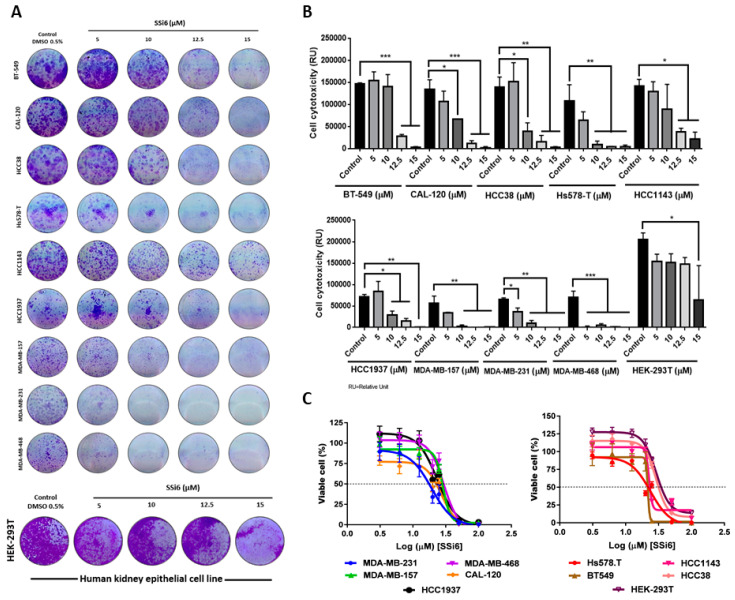
Cytotoxic effects of SSi6 on a panel of triple-negative breast cancer (TNBC) cells. (**A**) TNBC cells were seeded in 12-well plates and treated with indicated concentrations for 10 days steadily. (**B**) Graphs represent the relative cytotoxicity unit of SSi6 in TNBC cells. HEK-293T is a human embryonic kidney cell line, used for comparations of the cytotoxic effect of SSi6 in non-tumor human cells. The data represent the mean ± standard deviation of three independent tests in triplicate. The results were compared using one-way ANOVA, followed by Bonferroni’s analysis; * *p* < 0.05, ** *p* < 0.001, *** *p* < 0.0001. Effects of SSi6 on cell viability (CellTiter, MTS). Cells were plated in 96-well plates and incubated with different concentrations of SSi6, the vehicle was treated with 0.5% DMSO for 72 h. (**C**) IC_50_ plot on TNBC and HEK-293T cell lines treated with SSi6 concentrations. Data show mean ± SD of three independent experiments.

Colony assays were carried out to determine the cytotoxic activity of SSi6 in different TNBC cell lines. In this case, the cells were maintained with the treatment for 10 days to determine if those lines are resistant to the treatment and consequently can grow and remain viable. Thus, and considering the results presented in Figure 1A,B, it was observed that the number of colonies significantly decreased in all cell lines (mainly in the highest concentrations) in comparison with the negative control (non-tumor cells, HEK-293T). It is important to highlight that the cytotoxic effects of SSi6 are most significant on MDA cell lines (MDA-MB-157, MDA-MB-231, and MDA-MB-468) showing sensitivity at all concentrations tested (5, 10, 12.5, and 15 µM).

To complement these results a viability assay was performed to determine the IC_50_ values of SSi6 on TNBC cells. In this case, SSi6 treatment caused a more intense decrease in cell viability in MDA-MB-231 in comparison with other TNBC cell lines and the non-tumor cells (HEK-293T) (Figure 1C), with IC_50_ of 14.5 and 30.2 µM for MDA-MB-231 and HEK-293T cell lines, respectively, and a selectivity index (SI) of 2.08 (Table 1). These results are in agreement with those reported in our previous work, in which SSi6 presented higher cytotoxic activity against MDA-MB-231 (IC_50_ = 22.90 ± 0.35 μM) compared to the non-tumor breast cells MCF-10A (IC_50_ = 34.17 ± 2.49 μM), with a SI of ~1.49 for tumor cells after 48 h treatment [22]. Therefore, SSi6 had a greater cytotoxic effect on the MDA-MB-231 cell line compared to other TNBC cells tested.

### 3.2. SSi6 Doses No Provoke Toxic Effects on FVB Mice

**Figure 2:**
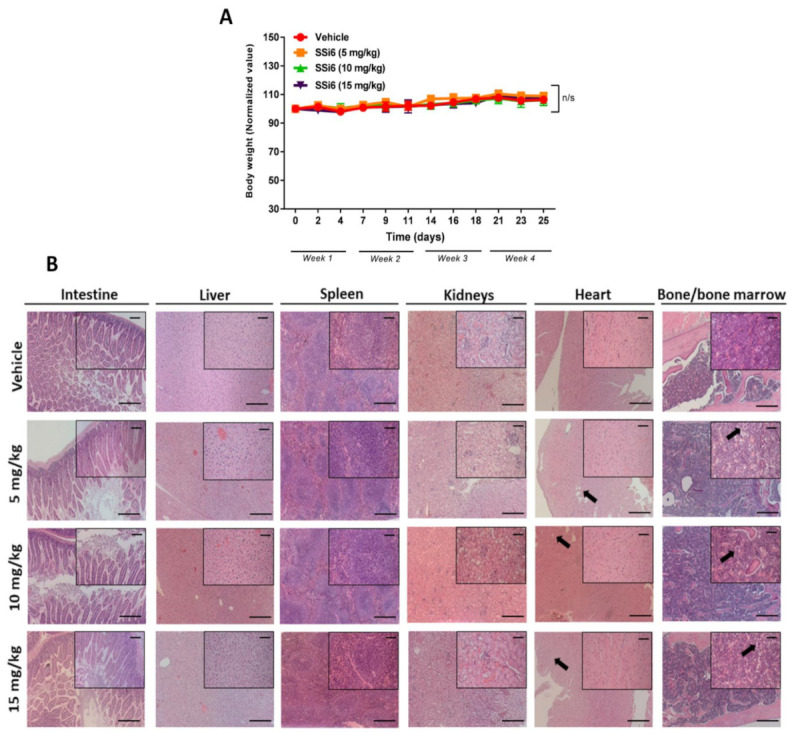
*In vivo* acute toxicity and tolerance effects of SSi6 doses on FVB mice. FVB female mice were randomly assigned to 4 groups (n = 5/per group) and treated with different doses of SSi6 (5, 10, and 15 mg/kg) and vehicle. During the experiment, body weight was monitored from the start of the experiment until the day of euthanasia. (**A**) Normalized body weight: vehicle group and SSi6 15 mg/kg started with different body weight from the start of treatment. (**B**) Representative photomicrographs of H&E-stained intestine, liver, spleen, kidney, heart, and bone/bone marrow of the female mice were taken. Arrows indicate some foci of subendocardial adipose tissue infiltration into the heart and some hyposegmented megakaryocytes in bone marrow. Images were analyzed and captured with the ZEISS AXIO Imager.M2 microscope, scale bar = 200 µm (low magnification) and 50 µm (high magnification).

Before clinical investigations of new compounds with promising *in vitro* results, exhaustive experiments are necessary in order to test their effectiveness and safety. In this sense, preclinical tests using animal models are extremely relevant to determine the doses to be applied, which should be the least toxic possible, but still effective [27]. Thus, the acute toxicity of different doses (5, 10, and 15 mg/kg) of SSi6 on female FVB mice was evaluated in terms of weight, hematological parameters, morphological alterations in various organs, and deaths during experiment. No significant adverse effects at any tested dose of SSi6 (even at 15 mg/kg) on weight loss (Figure 2A), and no deaths were observed.

Other parameters were considered to identify signs of toxicity in specific organs, such as an increase in the weight/size of organs (i.e., megaly), blood cell, and histopathology alteration signs. Several organs were collected and weighed at the end of the experiment to verify megalies that could be caused by the SSi6 treatment (Figure S5A–F), and no significant increase in the weight/size of the organs in SSi6-treated animals was observed when compared to the control group (vehicle), except for the kidneys (* *p* < 0.05) at 5 mg/kg of SSi6 (see Figure S5C). Furthermore, in the peripheral blood smear analyses, no significant changes in blood cells (i.e., basophils, eosinophils, neutrophils, platelets, and red blood cells) were observed (Figure S5G). Regarding the pathology analyses using H&E staining, no significant morphological/pathological alterations in the intestine, liver, spleen, kidneys/glomeruli, heart, and bone/bone marrow were observed between SSi6-treated animals and the vehicle group (Figure 2B). However, small changes in heart (little subendocardial adipose infiltration) and bone marrow (some hyposegmented megakaryocytes) were found in some mice from both groups (see black arrows in Figure 2B).

On the other hand, no clinical signs of systemic toxicity such as diarrhea, ataxia, spasms, bleeding, vomiting, dyspnea, lethargy, hypoactivity, sweating, and appearance of spots or alopecia, or neurological alterations were observed after the administration of SSi6 doses (4 weeks of the experiment). Moreover, no changes in the color or morphology of liver, spleen, heart, bone, intestine, and kidneys were observed in SSi6-treated mice (macroscopical observations of organs; data not shown). Taken together, these results show that SSi6 has relatively low acute intraperitoneal toxicity on mice and were relevant to choose the appropriate doses to be used in the xenograft studies, as described below.

### 3.3. SSi6 Retards Tumor Growth and Metastatic Spontaneous Progression of MDA-MB-231 Cells

An orthotopic xenograft model was performed to determine the activity of SSi6 on the inhibition of the primary tumor, in addition to the effect on possible spontaneous metastases. Different from the *in vitro* experiments, xenograft models were carried out using a modified TNBC line (MDA-MB-231/Luc) to evaluate the presence of metastases by bioluminescence (*in vivo* and *ex vivo*).

**Figure 3:**
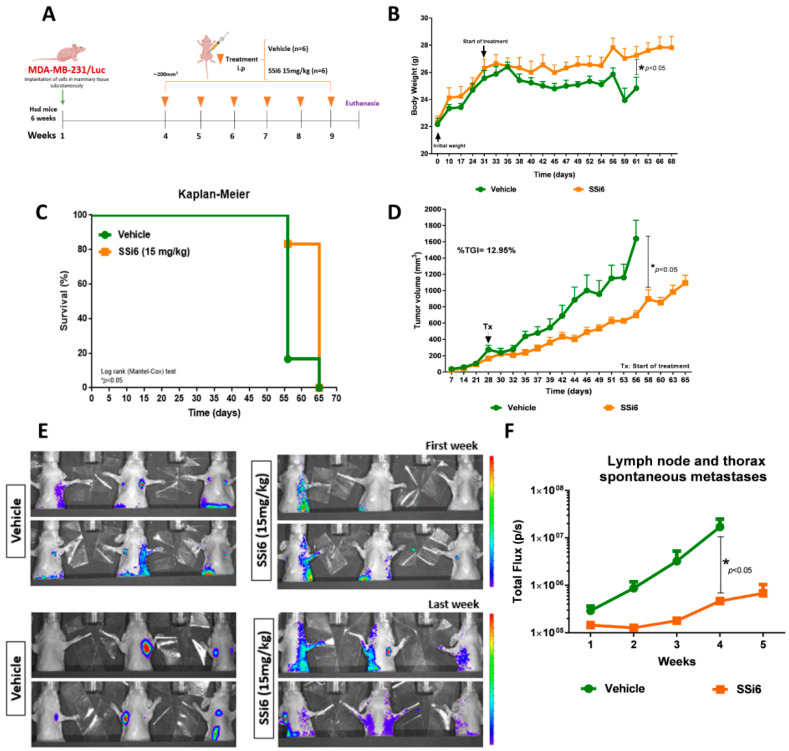
Effects of SSi6 dose on tumor development and axillary lymph node and thorax metastasis in an orthotopic xenograft model. (**A**) MDA-MB-231/Luc cells (1 × 10^6^) were injected into right and left mammary tissue subcutaneously of each 6-week-old female Athymic Nude mice. When the volume of the tumors reached 200 mm^3^, the mice were randomly divided into two groups (six mice in each group). Then, vehicle (90% physiological serum, 5% DMSO, and 5% Tween80) and SSi6 15 mg/kg were intraperitoneally injected daily in mice. (**B**) Body weight measured thrice a week since initial day from the final of an experiment. * *p* < 0.05; Student’s *t*-test. (**C**) Kaplan–Meier survival curve of mice treated with either vehicle or SSi6 15 mg/kg. Survival was calculated from the time of treatment initiation. Statistical differences (* *p* < 0.05) between the group treated with SSi6 dose and the vehicle group (log-rank Mantel-Cox test). (**D**) The volume of each tumor was measured three times a week for 65 days. Tumor growth inhibition (TGI) was calculated Tf final time point (volume) and T0 initial time point (volume) of SSi6 and vehicle group. TGI exerted by SSi6 12.95%. (**E**) Representative images of vehicle and SSi6 group with lymph node and lung metastases at first week until last week of treatment. (**F**) Quantification of lymph node and thorax spontaneous metastases as measured by total bioluminescence flux in photons/seconds.

MDA-MB-231/Luc cells were surgically transplanted bilaterally and subcutaneously into the breast tissue, and treatments started when the volume of tumors reached ~200 mm^3^ (Figure 3A). The chosen SSi6 dose (15 mg/kg) is equivalent to 1.21 mg/kg in humans (human equivalent dosage; HED). This value can be also expressed as a function of the surface area of an average human (considering 60 kg and 1.62 m^2^), obtaining a value of 45 mg/m^2^ [28]. A decrease in animals’ weight in the vehicle group was observed when compared to the SSi6-treated group (Figure 3B). Importantly, the vehicle group (* *p* < 0.05) presented higher mortality than SSi6 group, as shown in the Kaplan–Meier survival curves (Figure 3C). Of note, two mice from the vehicle group died before reaching the humane endpoint of the primary tumor (1500 mm^3^). The remaining mice were euthanized when this value was reached. Additionally, it is important to highlight that in the SSi6-treated group, a partial reduction of ~12.95% in tumor volume growth was observed (Figure 3D). These results can be attributed to the already known cytotoxic activity of SSi6 on the MDA-MB-231 cells shown *in vitro* in this study, as well as in previous works by our group [21,22].

*In vivo* bioluminescence analyses were performed once a week to evaluate whether SSi6 doses could inhibit metastases in the axillary lymph nodes (locally advanced metastatic disease/stage III) and the thorax area (related to lung metastases) formed spontaneously from the primary tumor (Figure S6A). The vehicle group had a higher incidence of metastases formation in the axillary lymph nodes and thoracic area in comparison to the SSi6-treated animals. Besides, SSi6 caused an apparent blockade of metastasis progression from axillary lymph nodes to the thorax area, which includes lungs (Figure 3E). In contrast, this progression was no prevented in the vehicle group (Figure 3F).

**Figure 4:**
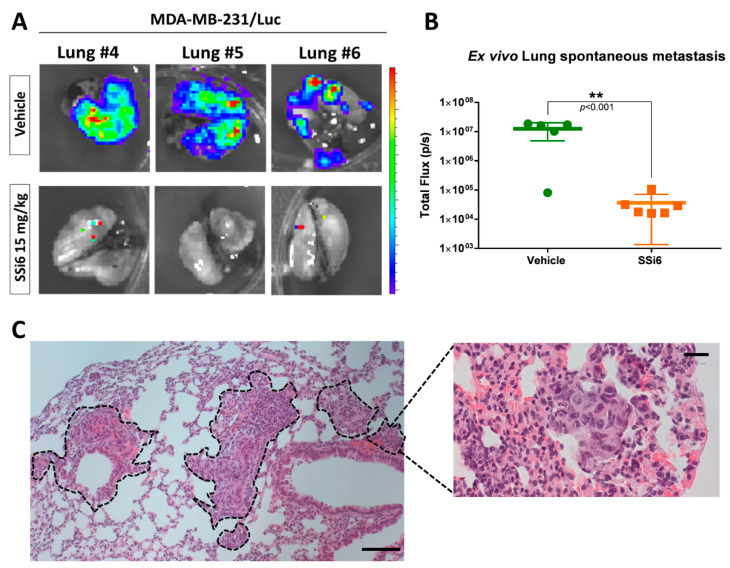

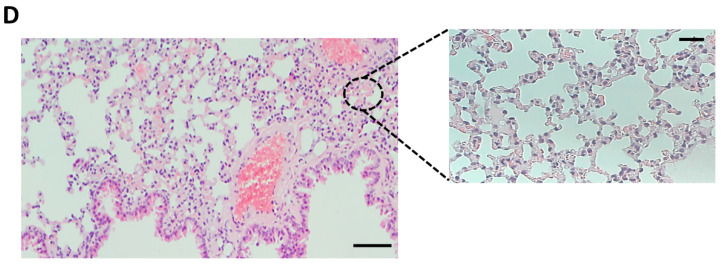
SSi6 treatment prevents spontaneous metastasis for lungs in an orthotopic model. MDA-MB-231 stably transduced with lentivector-luciferase was used to track cells by bioimaging after surgical transplantation into the mammary tissue of athymic nude mice. (**A**) Representative *ex vivo* bioluminescence images of lung from the vehicle and SSi6 15 mg/kg-treated mice. (**B**) Quantification of total flux at study endpoint. *p*-values were generated by Student’s *t*-test (* *p* < 0.05, ** *p* < 0.001). H&E-stained lung with areas marked with tumor growth of (**C**) vehicle and (**D**) SSi6 group. Images represent results in each treatment group, scale bar = 200 µm (low magnification) and 50 µm (high magnification).

To complement the metastatic progression results, *ex vivo* bioluminescence analyses were carried out in different vital organs, such as lungs, spleen, liver, and kidneys. Spontaneous metastases were confirmed mainly in the lung of 83% of the mice in the vehicle group (Figure 4A), but not in other organs (spleen, liver, and kidneys; Figure S6B). Differently, the SSi6-treated group had a negligible percentage (~1%) of metastases in the lung (Figure 4B), which confirms the inhibition of metastases progression from the lymph nodes towards this area, in addition to the lower evolution of metastases to other organs (see Figure S6B). These results are consistent with those obtained by histology analyses of the lungs, where mice of the vehicle group had multiple areas with metastatic foci (Figure 4C) compared to the SSi6 group that did not present metastases (Figure 4D).

Hematologic parameters were analyzed in both groups, as shown in Table 2. The vehicle group presented neutrophilia and lymphopenia, while SSi6 caused thrombocytopenia (highlighted in bold). As a complementary analysis, a peripheral blood smear and bone marrow histology were performed to determine whether the lung metastases could have infiltrated the bone marrow [29,30]. This experiment can also provide information about the changes in the levels of hematopoietic cells, which can be caused by induced cancer (as in this case) or by a collateral effect of the tested compound [30]. In both groups, no significant alterations in bone marrow cells were found (Figure S7A), and a good myeloid and erythroid ratio was observed, as well as good cellularity in peripheral blood (Figure S7B).

### 3.4. SSi6 Blocks the Progression of the Multi-Organ Metastases: Xenograft Model with Tumor Resection

Surgery is an important intervention that provides a chance for a cure for patients with any type of cancer [31]. In contrast, many studies suggest that surgical resection of the primary tumor is associated with an increased risk of accelerated growth of the metastatic disease and a greater formation of new metastatic foci since during this process there is a tissue injury leading to an acute inflammatory process [32].

In other to evaluate this situation, we applied a metastasis model with resection of the primary tumor to analyze the efficiency of SSi6 on the inhibition of multi-organ metastases (towards lung-heart, spleen, liver, and kidneys) and in the regression of primary tumor-derived from the surgery process. The primary tumor was resected with around 500 mm^3^ (after 7 weeks) after the initial metastases were formed (e.g., in the axillary lymph nodes). This condition was chosen to properly evaluate the effect of SSi6 on the inhibition and progression of metastases to other organs. The previous xenograft model (without resection) showed the effect of SSi6 on the partial inhibition of the primary tumor and the spontaneous metastases to the lungs (see Figure 4A,B) and other organs (see Figure S6B). However, this second model (with tumor resection) is focused mainly on the inhibition of the multi-organ metastases.

**Figure 5:**
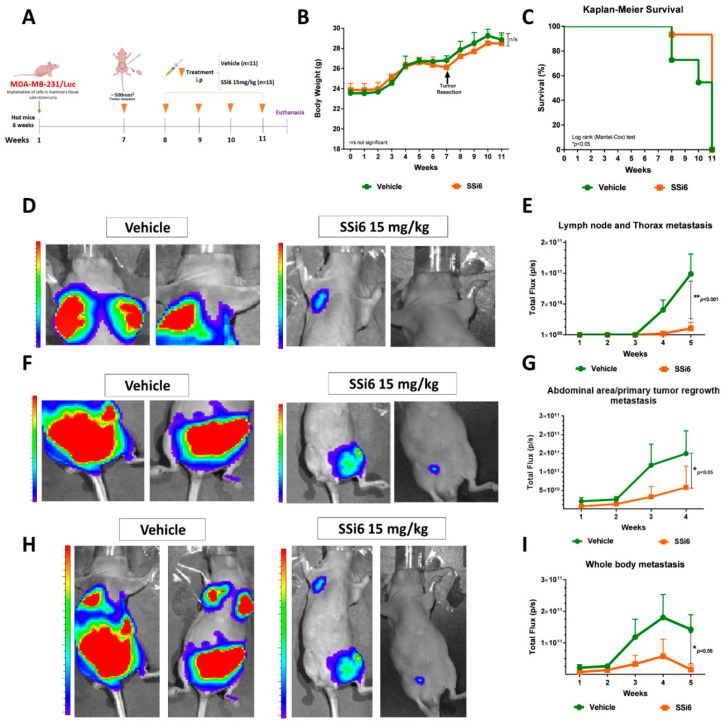
SSi6 increases survival, reduces whole-body metastases and tumor regrowth, after resection of the primary tumor. (**A**) The TNBC MDA-MB-231/Luc tumor was removed with a volume of approximately 500 mm^3^ in both groups (vehicle n = 11; SSi6 15 mg/kg n = 15) after treatment was started daily by intraperitoneal route and ended when the mice showed signs of distress. (**B**) Body weight was monitored three times a week, before and after surgical resection of the primary tumors. (**C**) Kaplan–Meier Survival curve from the vehicle and SSi6-treated mice. *P*-value was calculated by log-rank Mantel-Cox test. (**D**) Representative images of bioluminescence and (**E**) quantification of the upper segment (axillary lymph nodes and thoracic region), (**F**) lower segment, and (**G**) quantification showing the abdominal region and the regrowth of primary tumors, besides (**H**) whole-body images and (**I**) quantification obtained from the sum of each segment of the treated groups (vehicle and SSi6).

SSi6 (15 mg/kg) was administered daily after one week of resection of the primary tumor (Figure 5A). This dose did not represent any toxicity in mice since no changes during the monitoring of bodyweight from the beginning to the end of the treatment were observed (Figure 5B). Additionally, the Kaplan–Meier curves (Figure 5C) show that the SSi6-treated group had a higher percentage of survival compared to the vehicle group (* *p* < 0.05).

During the experiment, metastases were developed more markedly in the vehicle group firstly on axillary lymph nodes and thorax (lung) (Figure 5D,E). Consistent with previous observations, we found that the SSi6 group exhibited fewer metastases in the abdominal area and primary tumor regrowth (Figure 5F,G). Moreover, a high frequency of metastases was observed in the vehicle group (Figure S8A) in comparison to SSi6-treated group (Figure S8B).

**Figure 6:**
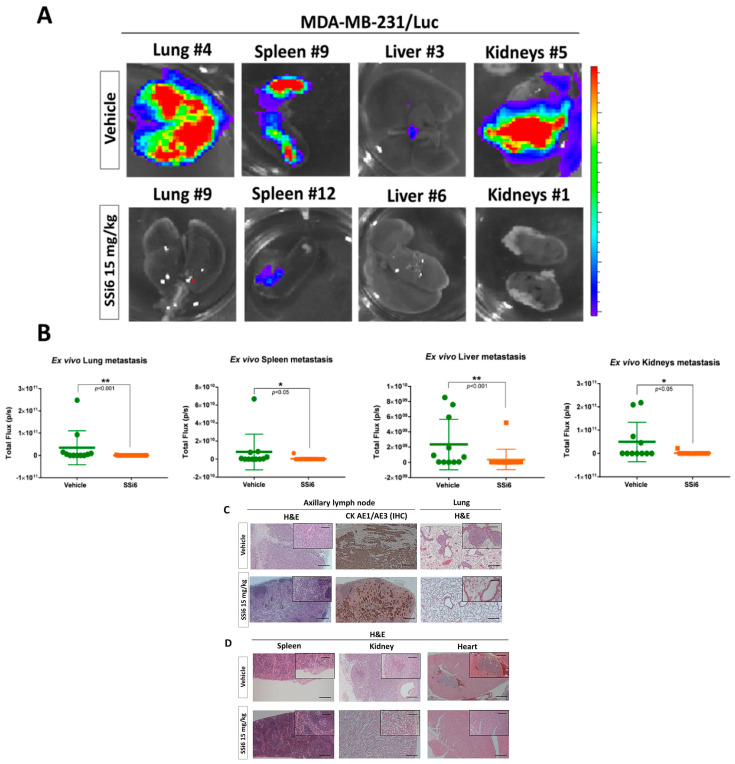
SSi6 interferes with multi-organ metastases and block the advancement of MDA-MB-231/Luc cells from lymph nodes to the lungs in a xenograft model with resection of the primary tumor. (**A**) *Ex vivo* bioluminescence images of the organs of the vehicle group (n = 11) and SSi6 15 mg/kg (n = 15). (**B**) *Ex vivo* graphs of the lung, spleen, liver, and kidneys, respectively. *p*-values were generated by Student’s *t*-tests (* *p* < 0.05, ** *p* < 0.001). (**C**) Micrograph of H&E staining and anti-human pan-cytokeratin IHC of harvested tissue to confirm the presence of breast cancer cell in the axillary lymph node. (**D**) Representative images of H&E stains showing metastatic nodules in the spleen, kidneys, and heart of the treated groups.

Furthermore, SSi6 treatment significantly decreased the quantification of whole-body bioluminescence (Figure 5H,I) and the number of mice with metastases (Figure S8C). As expected, *ex vivo* bioluminescence (Figure 6A) confirmed metastases in most organs evaluated, such as lungs (~45%), spleen (~36%), and kidneys (~36%) (Figure 6B; ** *p* < 0.001, * *p* < 0.05) in some of the vehicle-treated mice. Besides, *ex vivo* images of all organs at the final of the experiment visually show a higher metastatic burden in mice from the control group, compared to SSi6-treated mice group (Figure S9; vehicle n = 11, SSi6 n = 15).

Next, H&E stains of axillary lymph nodes and lungs showed that SSi6 treatment inhibited the progression of locally advanced metastatic diseases from lymph nodes to the lungs, contrary to the observed in the vehicle group, which was also confirmed by IHC (Figure 6C). Finally, according to the *ex vivo* bioluminescence images, metastases to other vital visceral organs were almost totally inhibited in the SSi6-treated group, where no area/foci of metastatic growth were observed (Figure 6D).

Conjointly, the results presented here provide strong evidence that the xenograft models with locally advanced disease, with and without resection of the primary tumor, generate simultaneous metastases, and, more importantly, that SSi6 has a promising ability to inhibit metastatic progression, mainly from the axillary lymph nodes to the lungs, as well as to prevent multi-organ metastases.

## 4. Discussion

TNBC is a heterogeneous group of aggressive tumors that exhibit higher rates of relapse and shorter survival compared to other breast cancer subtypes [33,34]. In the absence of well-defined molecular targets, standard chemotherapy is widely used to treat metastatic TNBC [35]. However, the safety and toxicity of this therapy to normal tissues remain primary concerns. Therefore, the search for new compounds (natural or synthetic) with more selective and fewer collateral effects compared to the actual chemotherapy drugs is one of the biggest challenges in cancer treatment. In this context, it is essential to test the efficacy of new compounds and to incorporate them into preclinical models that evaluate heterogeneous populations of cells derived from human tumors.

As is already known, gingerols (compounds present in ginger roots) are the compounds that constitute the rhizome of the plant, to which the biological/pharmacological properties are attributed [36,37]. Previously, we have shown evidence of the effectiveness *in vitro* of SSi6 to induce cell death in the MDA-MB-231 cell line. In this study, we show SSi6 potentiated selective cytotoxic effects in the triple-negative line (MDA-MB-231). Unlike the original natural compound (6G), SSi6 induced cell death by activation of two path-ways, autophagy and caspase-independent apoptosis, promoted by the induction of reactive oxygen species. This strategy of inducing distinct mechanisms of cell death could be used in triple-negative tumors resistant to drugs that induce canonical apoptosis [22]. We wanted to extend this study with the use of preclinical models that would allow determining the therapeutic efficacy of this new compound. It is worth mentioning that there are no previous studies that demonstrate the antitumor and antimetastatic effects of the SSi6 *in vivo*. Therefore, this work was compared with other *in vitro* and *in vivo* studies that demonstrated the therapeutic role of other no-modified gingerols, such as 6G and [10]-gingerol.

Xu et al. [38] demonstrated the cytotoxic potential of 6G at high concentrations (10, 30, and 50 µM) in renal carcinoma cell lines. Authors demonstrated that 6G inhibited colony growth in a concentration-dependent manner (in 7 days of treatment) in ACHN, 786-O, and 769-P cell lines, mainly at 30 and 50 µM. Furthermore, another study by Luo and co-workers [39] reported the effect of 6G on HGC-27 human gastric carcinoma, treated with 300 µM of 6G together with ionizing radiation to sensitize the cells and improve the activity of this compound. Despite the radiosensitization received, the IC_50_ value remained high (386.3 µM) after 48 h of 6G treatment. Our study clearly shows that SSi6 is more active than its unmodified analogous 6G since it presented better cytotoxic activity, demonstrated by the inhibition of colony formation at low concentrations in the TNBC lines (MDA-MB-231, MDA-MB-157, and MDA-MB-468). Furthermore, the calculated IC_50_ values were significantly lower, also demonstrating the selectivity of SSi6 against the MDA-MB-231 cell line.

It has been reported in several studies that 6G has no acute toxicity in animal models. Rastogi et al. [40] demonstrated that 6G doses of 2.5 and 5 mg/kg did not alter the bodyweight of athymic mice in a xenograft model with cervical tumor HeLa cells. In the same study, alanine aminotransferase enzyme, and aspartate aminotransferase analyses concluded that 6G is not hepatotoxic to the animals. Consistent with the above results, our work showed similar results when analyzing histopathology and blood parameters, showing that SSi6 does not cause toxic effects in FVB mice even at 15 mg/kg. This suggests that the modification on the molecular structure of 6G to produce SSi6 has not an effect on the acute toxicity of the compound, but is very important to increase its selectivity and cytotoxic effects towards TNBC cells.

Regarding the first xenograft model (without tumor resection) and as an important point of this model was that we allowed the installation/establishment of metastases in axillary lymph nodes and SSi6 treatment was initiated after the primary tumor reached a volume of ~200 mm^3^. Considering this idea, SSi6 showed good antitumor activity to retard/inhibit the continued growth of the primary tumor compared to the control group. More importantly, SSi6 inhibited the progression of spontaneous metastasis from axillary lymph nodes to the lung in most mice in the treated group (15 mg/kg), proving the excellent antimetastatic activity of SSi6. In contrast, most *in vivo* studies that evaluate antimetastatic effects of 6G use very high doses, probably due to its low *in vivo* activity. As demonstrated by Zhong et al. [41], only doses of 100 and 200 mg/kg of 6G (~10 times higher than used in this work) were able to suppress tumor progression and lung and liver metastases in a xenograft model using the MCF-7 breast cancer line. Nevertheless, different from our study, the treatment started when the tumor volume was approximately 10 mm^3^, that is when the metastatic disease did not reach an advanced stage in the lymph nodes.

On the other hand, to better mimic the clinical metastatic disease condition in women, in which the tumor is discovered, the surgical resection is performed, and the chemotherapy regimen is started, then we developed a preclinical xenograft model with resection of the primary tumor and started the SSi6 treatment. Previous studies by our research group showed the antimetastatic activity of [10]-gingerol (10G), another gingerol-derived compound [42,43]. In an orthotopic xenograft model, 10G (10 mg/kg) inhibited the primary tumor growth and circulating tumor cells (CTC) in mice injected with the MDA-MB-231 HMTL.6 line (isolated from spontaneous lung metastases) [43]. Inhibition of CTC was closely related to the settlement of the cells in the lungs (pulmonary metastasis). Here, we have provided evidence that this xenograft model with resection of the primary tumor also responds to treatment with SSi6, which was similarly effective in inhibiting visceral metastases (spleen, liver, and kidneys). Notably, we were able to prove that SSi6 inhibited metastatic progression from axillary lymph nodes to the lungs in most mice in the treated group.

## 5. Conclusions

In summary, the orthotopic xenograft model allowed us to determine that SSi6 has good antitumor activity *in vivo,* shown through partial inhibition of the growth of the primary tumor, without significant negative side effects on the weight or mortality of the mice. More importantly, in the second xenograft model, SSi6 treatment blocked the progression of metastasis from the lymph nodes to the lungs as well as to multiple organs, including typical visceral organ metastases of breast cancer. Finally, we provide preclinical evidence that SSi6 is effective as a single agent, which could potentially be used in clinical trials as adjunctive therapy for the treatment of TNBC.

## Figures and Tables

**Table 1:** IC_50_ values of SSi6 in human triple-negative breast cancer cell lines after 72 h of treatment.

IC_50_ ± SD * (µM)											
Compound	MDA-MB-231	MDA-MB-468	MDA-MB-157	CAL-120	HCC1937	Hs578.T	BT-549	HCC1143	HCC38	HEK-293T **	SI ***
SSi6	14.51 ± 2.4	29.57 ± 2.9	28.5 ± 0.8	27.9 ± 3.3	26.1 ± 1.9	23.1 ± 1.1	20.25 ± 0.6	30.6 ± 1.2	28.9 ± 1.9	30.2 ± 1.8	2.08

* Standard deviation ** Human embryonic kidney cell line *** Selectivity index = IC50HEK-293T/IC50MDA-MB-231.

**Table 2:** Hemogram of mice for each group. Altered values are highlighted in bold. *p*-values were generated by Student’s *t*-test, vehicle group vs. SSi6 group (* *p* < 0.05; ** *p* < 0.001).

Parameter	Vehicle Group	SSi6 Group	Normal Range	Unit	*p*-Value
WBC	5.78	5.78	0.80–10.60	10^9^/L	n/s
Neu#	**5.00**	2.36	0.23–3.60	10^9^/L	* *p* < 0.05
Lym#	2.28	2.82	0.60–8.90	10^9^/L	n/s
Mon#	0.38	0.35	0.04–1.40	10^9^/L	n/s
Eos#	0.17	0.22	0.00–0.51	10^9^/L	n/s
Bas#	0.05	0.04	0.00–0.12	10^9^/L	n/s
Neu%	**58.24**	39.17	6.5–50.0	%	* p < 0.05
Lym%	**32.58**	49.38	40.0–92.0	%	* *p* < 0.001
Mon%	6.38	6.48	0.9–18.0	%	n/s
Eos%	3.02	4.20	0.0–7.5	%	n/s
Bas%	0.88	0.77	0.0–1.5	%	n/s
RBC	8.41	8.16	6.50–11.50	10^12^/L	n/s
HGB	13.73	13.63	11.0–16.5	g/dL	n/s
HCT	39.65	40.92	35.0–55.0	%	n/s
MCV	47.13	50.07	41.0–55.0	fL	n/s
MCH	16.33	16.68	13.0–18.0	pg	n/s
MCHC	346.17	334.67	300–360	g/L	n/s
RDW-CV	14.27	16.10	12.0–19.0	%	n/s
RDW-SD	29.42	35.80	23.0–39.0	fL	n/s
PLT	756.33	**358.54**	400–1600	10^9^/L	** *p* < 0.001
MPV	5.77	5.93	4.0–6.2	fL	n/s

Abbreviations: WBC, white blood cells; RBC, red blood cells; HGB, hemoglobin; HCT, hematocrit; MCV, medium corpuscular volume; MCH, medium corpuscular hemoglobin; MCHC, mean corpuscular hemoglobin concentration; RDW-CV, Range of Distribution of Erythrocytes measured as Coefficient of Variation; RDW-SD, Range of Distribution of Erythrocytes measured as standard deviation; PTL, platelets; MPV, medium platelet volume.

## Data Availability

The data presented in this study are available on request from the corresponding author.

## Supplementary Materials

The following are available online at https://www.mdpi.com/article/10.3390/cancers13122855/s1, Figure S1: Chemical structures of semi-synthetic compounds, (**A**) SSi6-*trans* and (**B**) SSi6-*cis*, Figure S2: H NMR (400 MHz, CDCl_3_, 298 K) spectrum of (**A**) SSi6-trans and (**B**) SSi6-cis, Figure S3: Electron spray MS/MS mass spectrum (CHCl_3_, 298 K) of (**A**) SSi6-*trans* and (**B**) SSi6-*cis*, in positive mode, Figure S4: Comparison of the antitumor activity of SSi6 isomers, Figure S5: Acute intraperitoneal toxicity study, Figure S6: SSi6 dose inhibits spontaneous metastases in an orthotopic xenograft model, Figure S7: Complementary data of orthotopic xenograft model, Figure S8: Bioluminescent images of metastasis xenograft model with resection of the primary tumor, Figure S9: SSi6 treatment prevented lung and heart, spleen, liver, kidneys metastasis in a model with primary tumor resection.
