# Supplementary material for: [6]-Gingerol-Derived Semi-Synthetic Compound SSi6 Inhibits Tumor Growth and Metastatic Dissemination in Triple-Negative Breast Cancer Xenograft Models
Source: Cancers (Basel). 2021 Jun 8;13(12):2855. doi: 10.3390/cancers13122855 (PMC8228746; doi:10.3390/cancers13122855)
Supplement: Supplementary file 1 [file cancers-13-02855-s001.zip › cancers-1216702-supplementary.pdf]

cancers

MDPI

*Supplementary Files*

# [6]-gingerol-derived semi-synthetic compound SSi6 inhibits tumor growth and metastatic dissemination in triple-negative breast cancer xenograft models

**Liany Luna-Dulcey, James Almada da Silva, Veronica Jimenez-Renard, Eduardo Caleiras, Silvana Mouron, Miguel Quintela-Fandino and Marcia R. Cominetti**

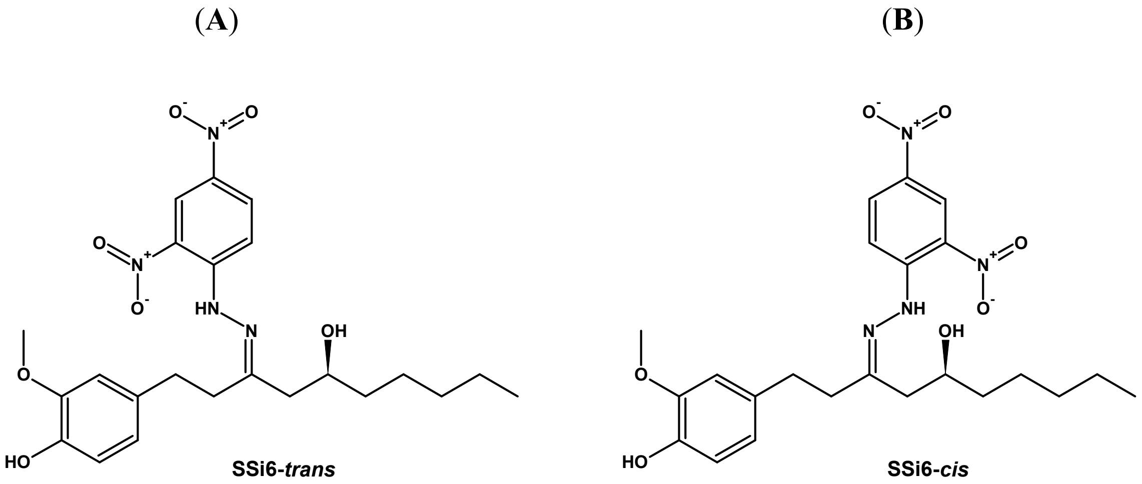


**Figure S1.** Chemical structures of semi-synthetic compounds, (**A**) SSi6-*trans* and (**B**) SSi6-*cis*.

(**A**)

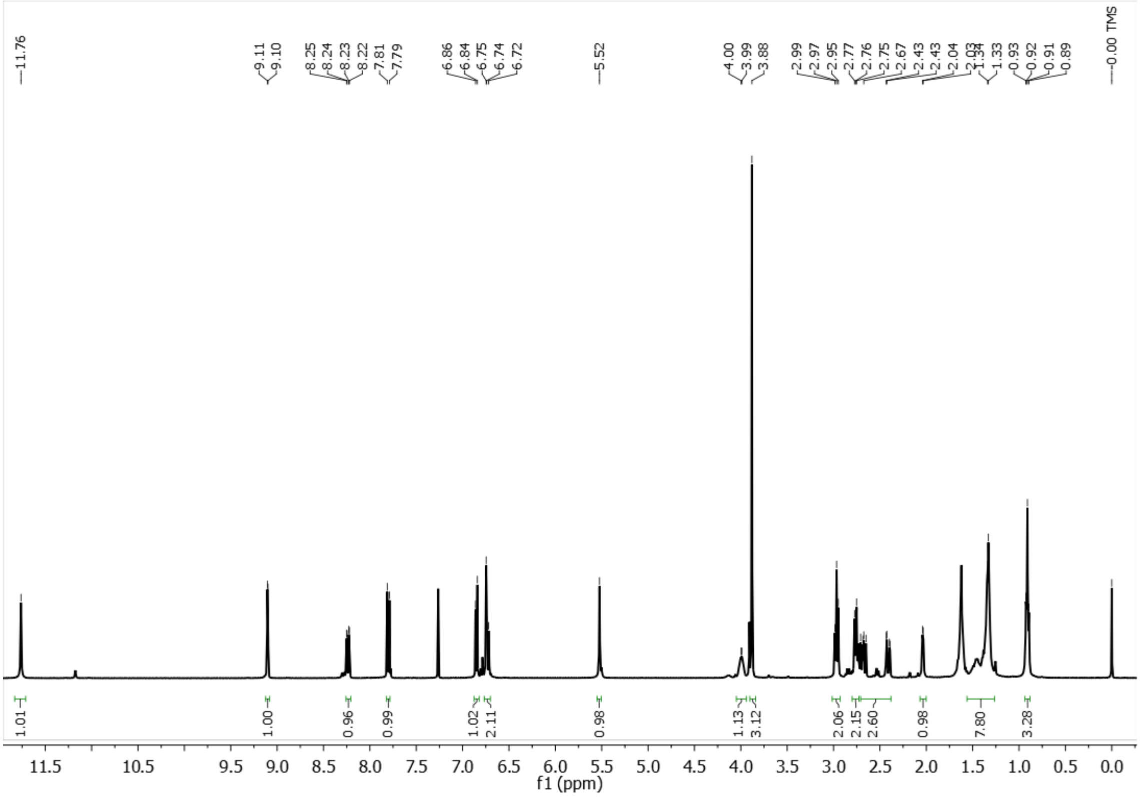


(**B**)

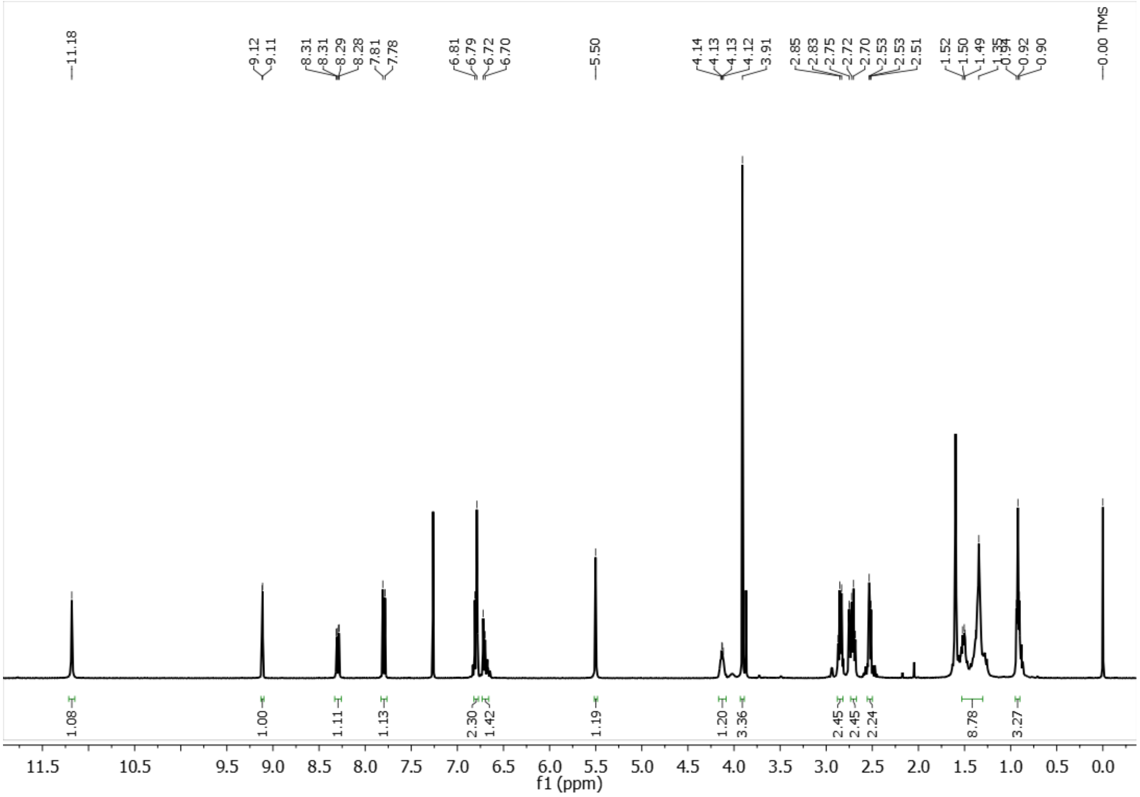


**Figure S2.** H NMR (400 MHz, $CDCl_3$, 298 K) spectrum of (**A**) SSi6-trans and (**B**) SSi6-cis.

(**A**)

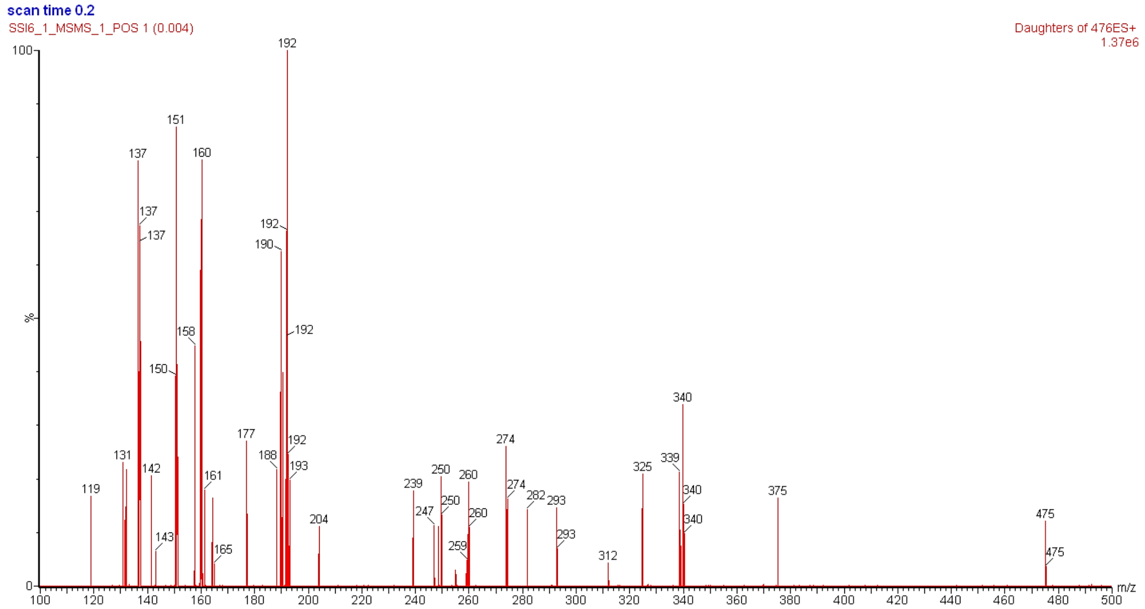


(**B**)

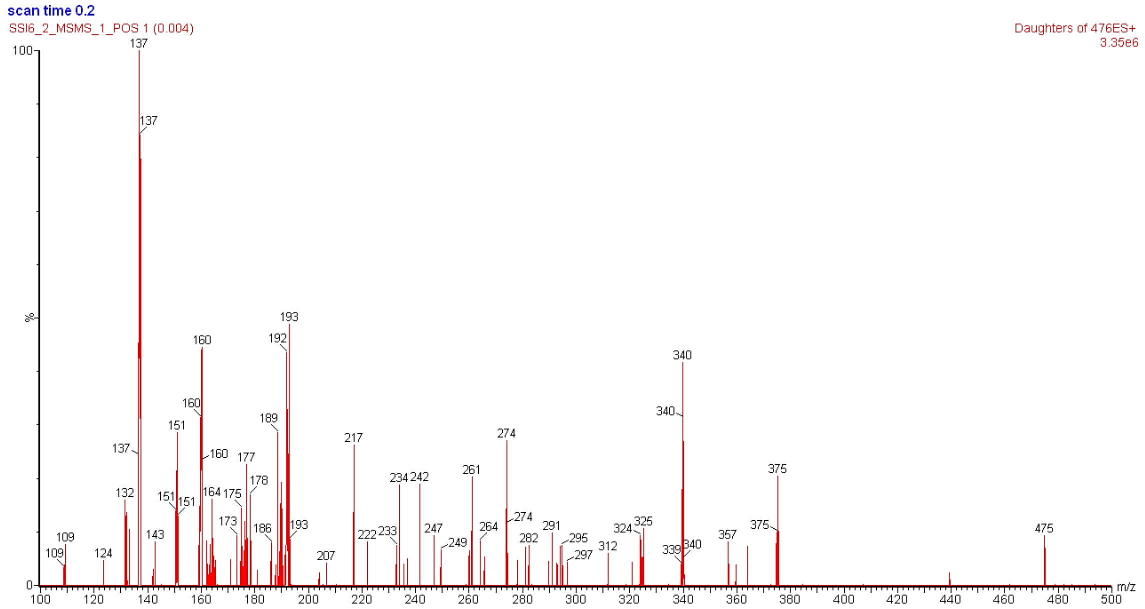


**Figure S3.** Electron spray MS/MS mass spectrum ($CHCl_3$, 298 K) of (**A**) SSi6-*trans* and (**B**) SSi6-*cis*, in positive mode. The molecular ion peak $MH^+$ was m/z 475 g mol$^{-1}$.

| **IC$_{50}$ (µM)** |
| --- |

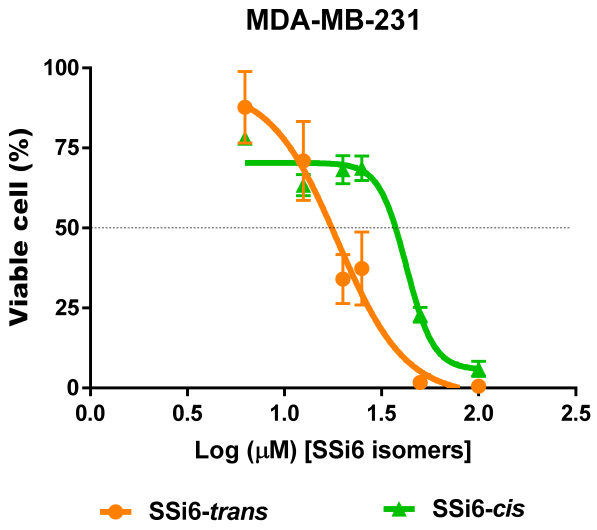


| Isomers | MDA-MB-231 |
|---|---|
| SSi6-*trans* | 14.51 ± 2.4 |
| SSi6-*cis* | 42.4 ± 1.25 |

**Figure S4.**Comparison of the antitumor activity of SSi6 isomers. MDA-MB-231 cells were treated (72 h) with increasing concentrations of SSi6 isomers using CellTiter assay. $IC_{50}$ values for triple-negative cell line are indicated in the panel to the side. Data show mean ± SD of three independent experiments.

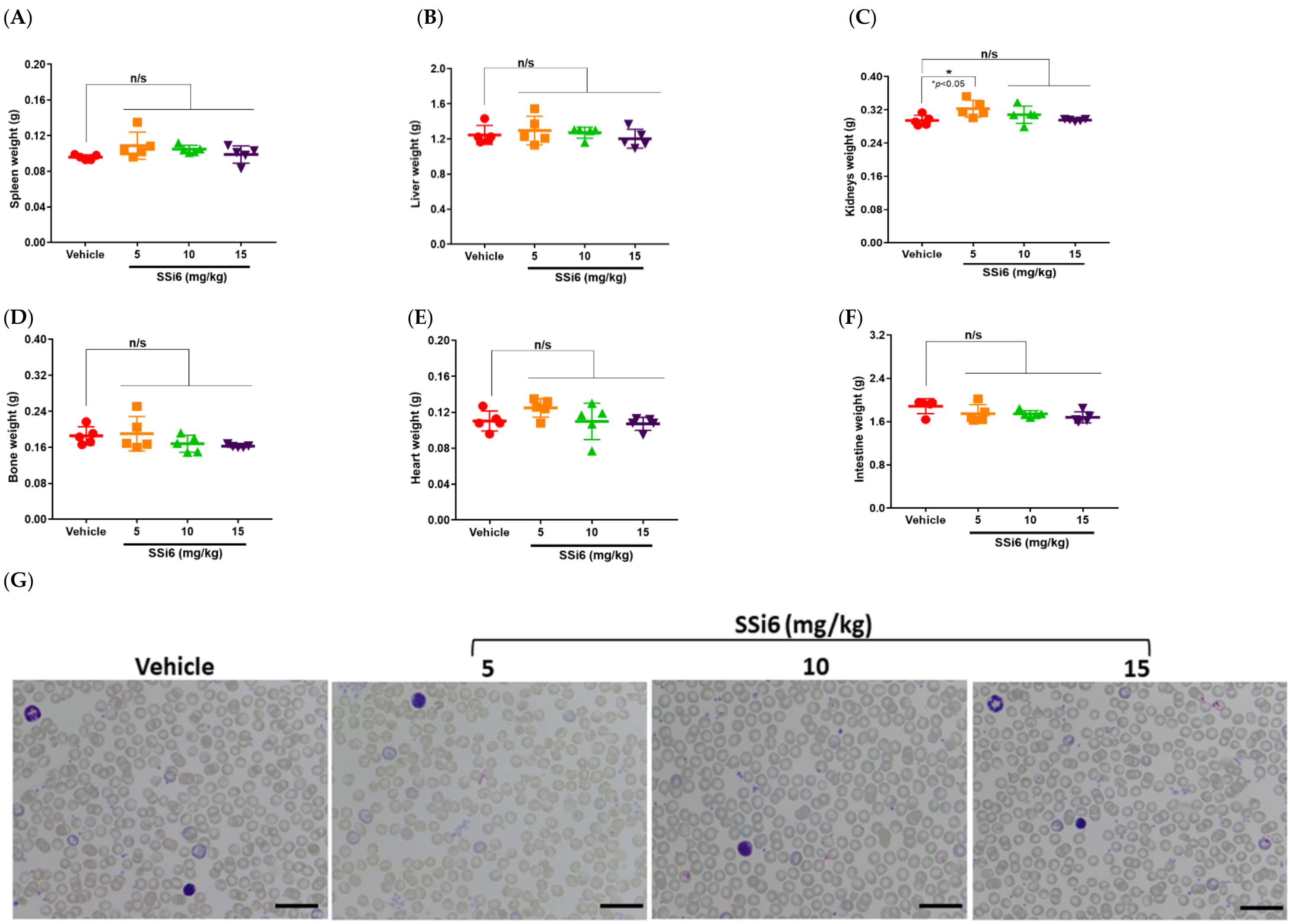


**Figure S5.** Acute intraperitoneal toxicity study. In euthanasia, the value of the weight of the organs as (**A**) spleen, (**B**) liver, (**C**) kidneys, (**D**) bone, (**E**) heart, and (**F**) intestine was recorded to discard any megaly caused by the treatment and collected for histopathological analysis. Results were compared using one-way ANOVA, followed by Bonferroni's post-hoc analysis. * $p < 0.05$ vs. control group (vehicle). (**G**) Photomicrographs of peripheral blood smears from female FVB mice that had received treatment with vehicle and SSi6 indicated doses, scale bar = 500 μm.

(**A**)

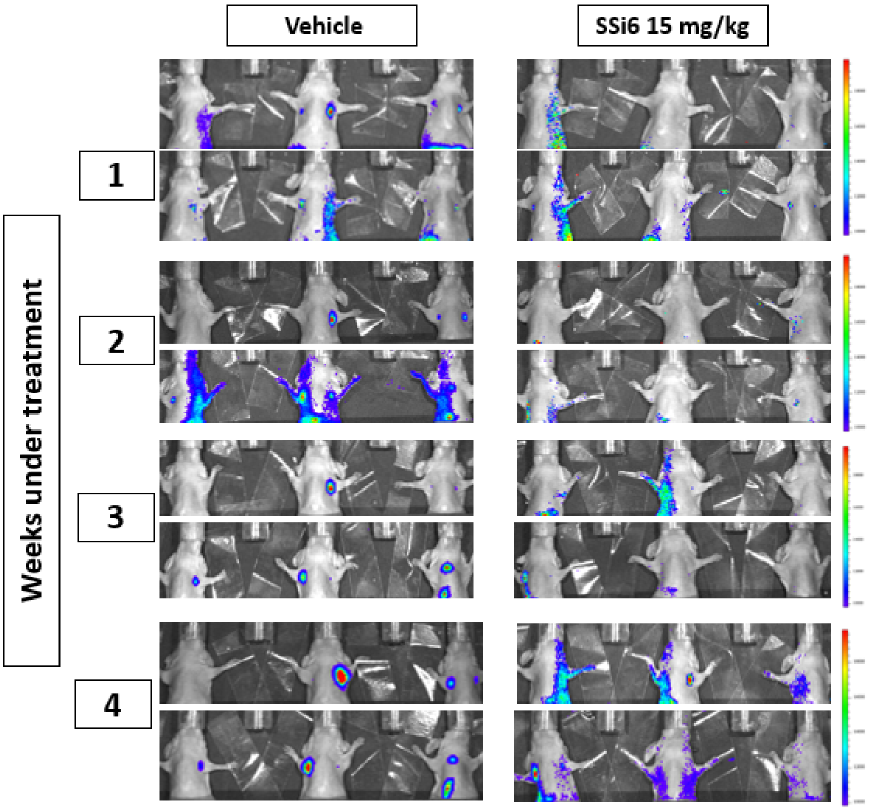


(**B**)

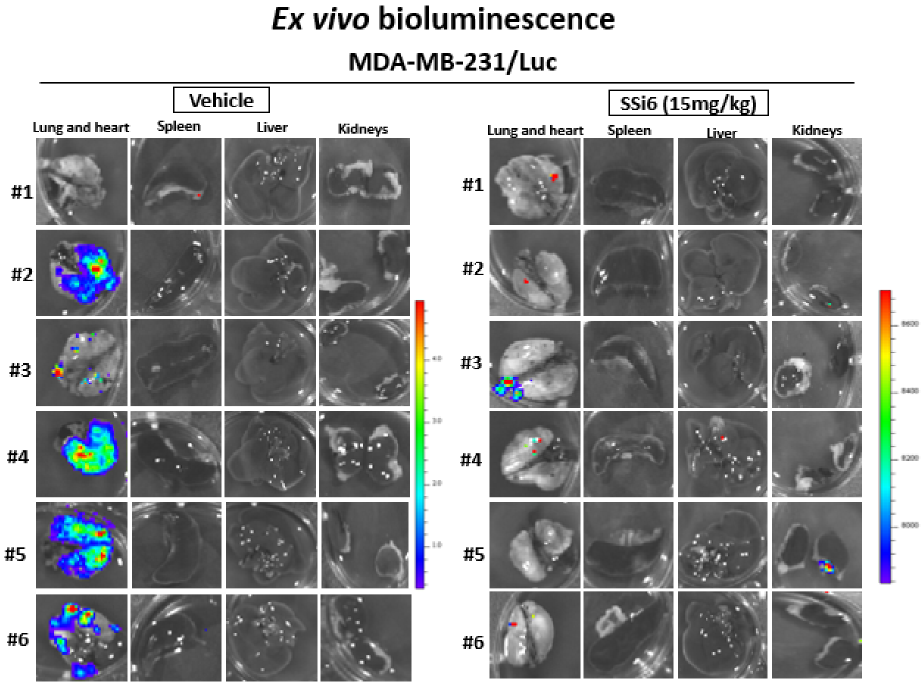


**Figure S6.** SSi6 dose inhibits spontaneous metastases in an orthotopic xenograft model. MDA-MB-231/Luc was surgically transplanted into mammary tissue of Hsd: Athymic Nude-Foxn1nu female mice. (**A**) Representative images of the vehicle and SSi6 group with lymph node and thorax metastases at weeks under treatment. Primary tumors were covered to allow visualization of small metastatic foci. (**B**) *Ex vivo* bioluminescence images of lung, spleen, liver, and kidneys of all mice in

the vehicle group and treated with SSi6 15 mg/kg. Quantification of total flux at study endpoint. *p*-values were generated by Student's *t*-test (* $p < 0.05$; ** $p < 0.001$).

(**A**)

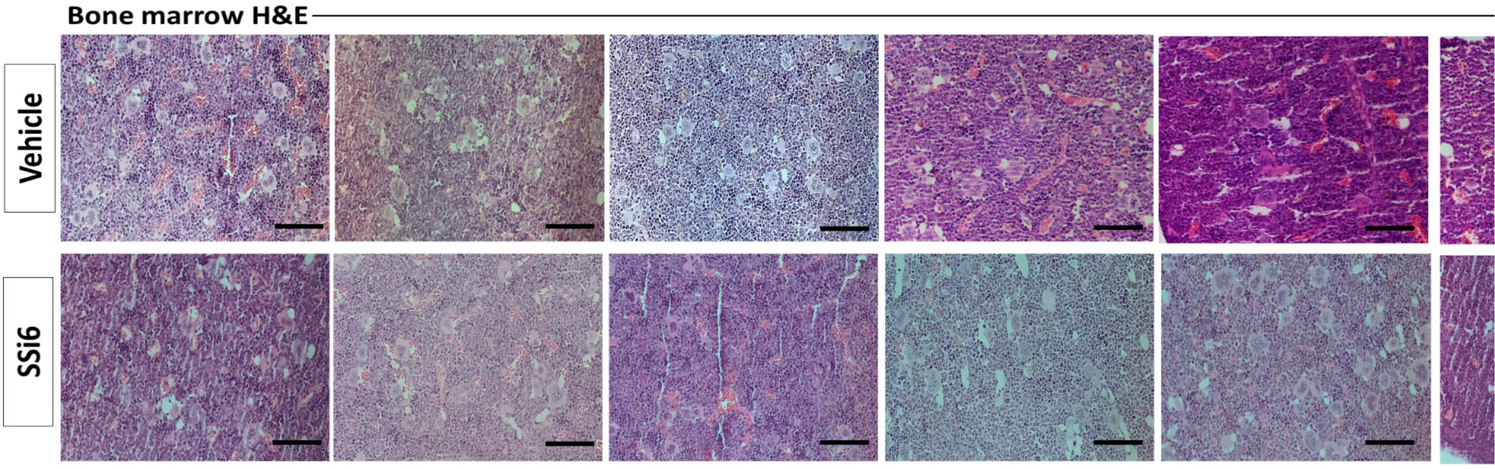


(**B**)

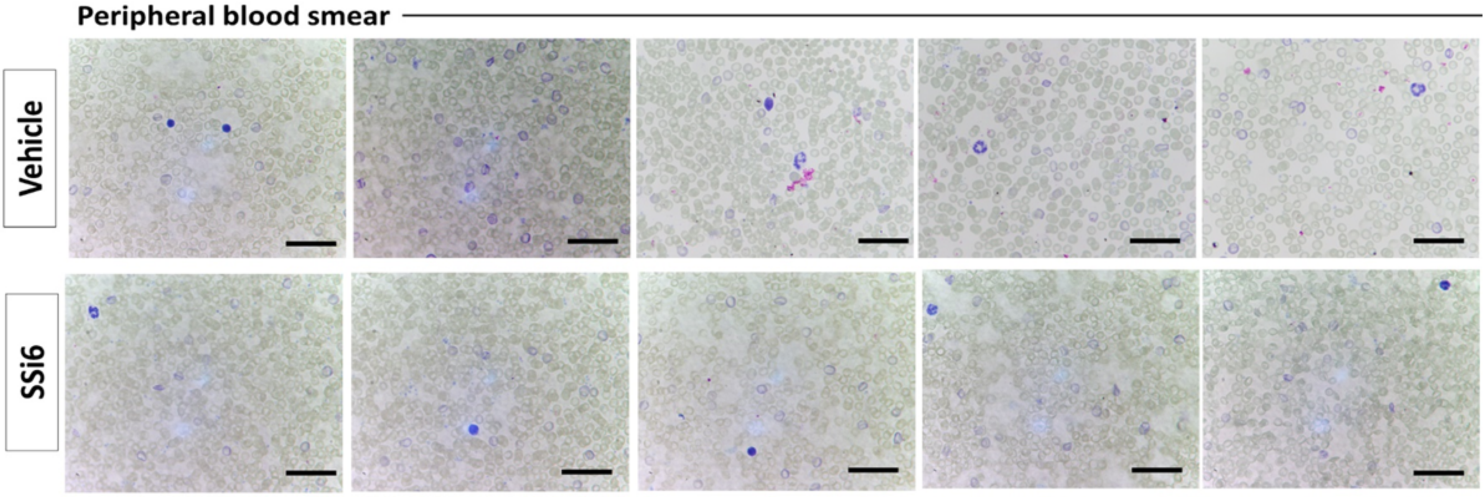


**Figure S7.** Complementary data of orthotopic xenograft model. (**A**) Photomicrographs of bone marrow (scale bar = 50 µm) from female athymic nude mice that had receives treatment with vehicle and SSi6 15 mg/kg. (**B**) Peripheral blood-smeared (scale bar = 25 µm) onto clean microscope slides, allowed to air-dry, then fixed with absolute methanol for 3 min and stained with May-Gründwald Giemsa and bone marrow was stained with hematoxylin and eosin (H & E).

(**A**)

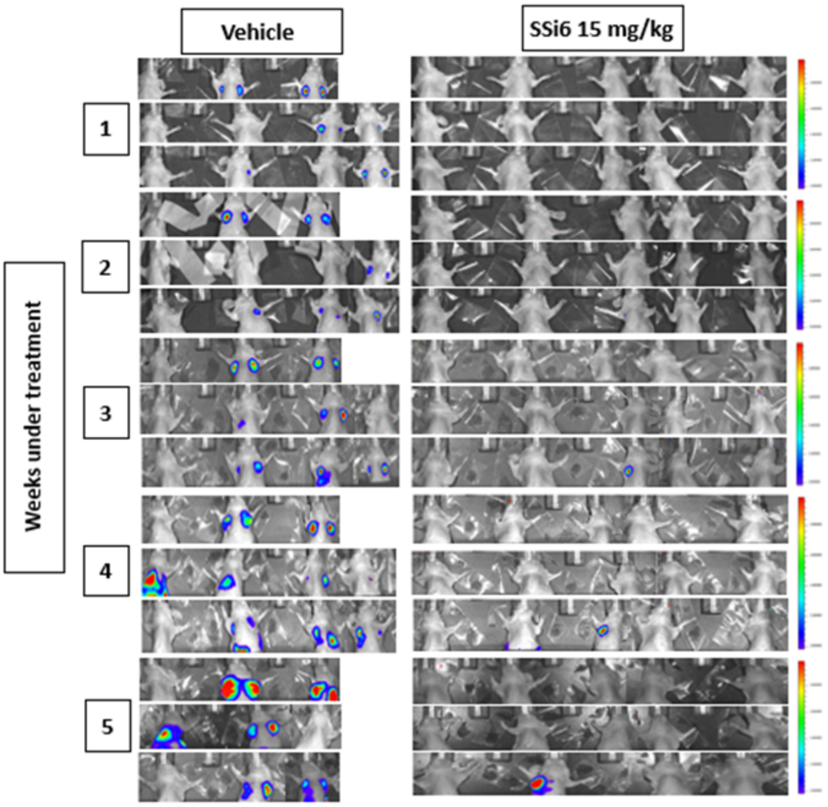

(**B**)

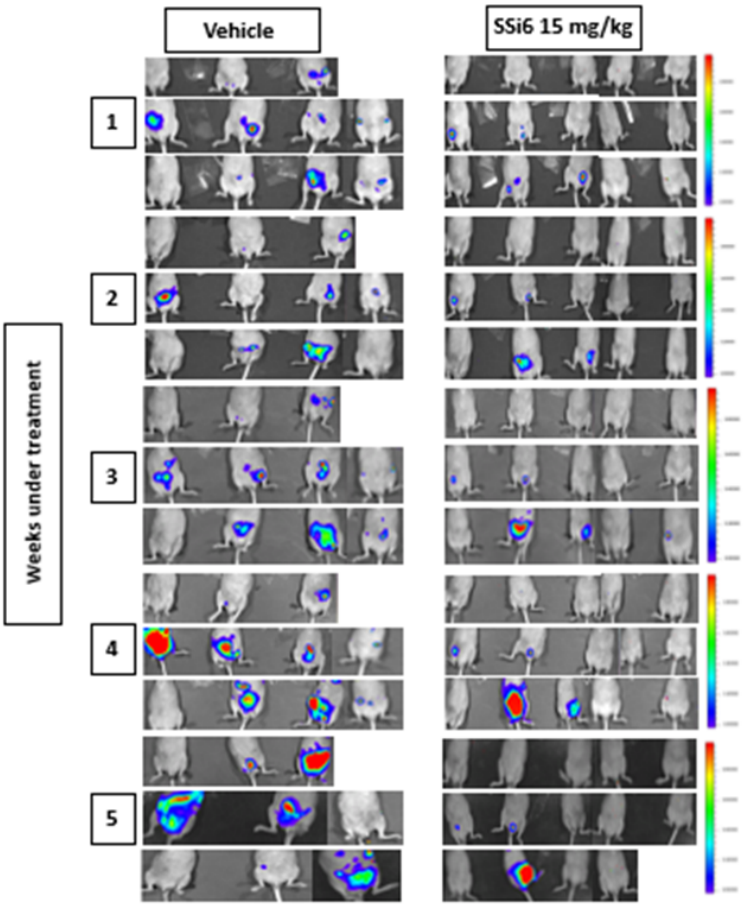

(C)

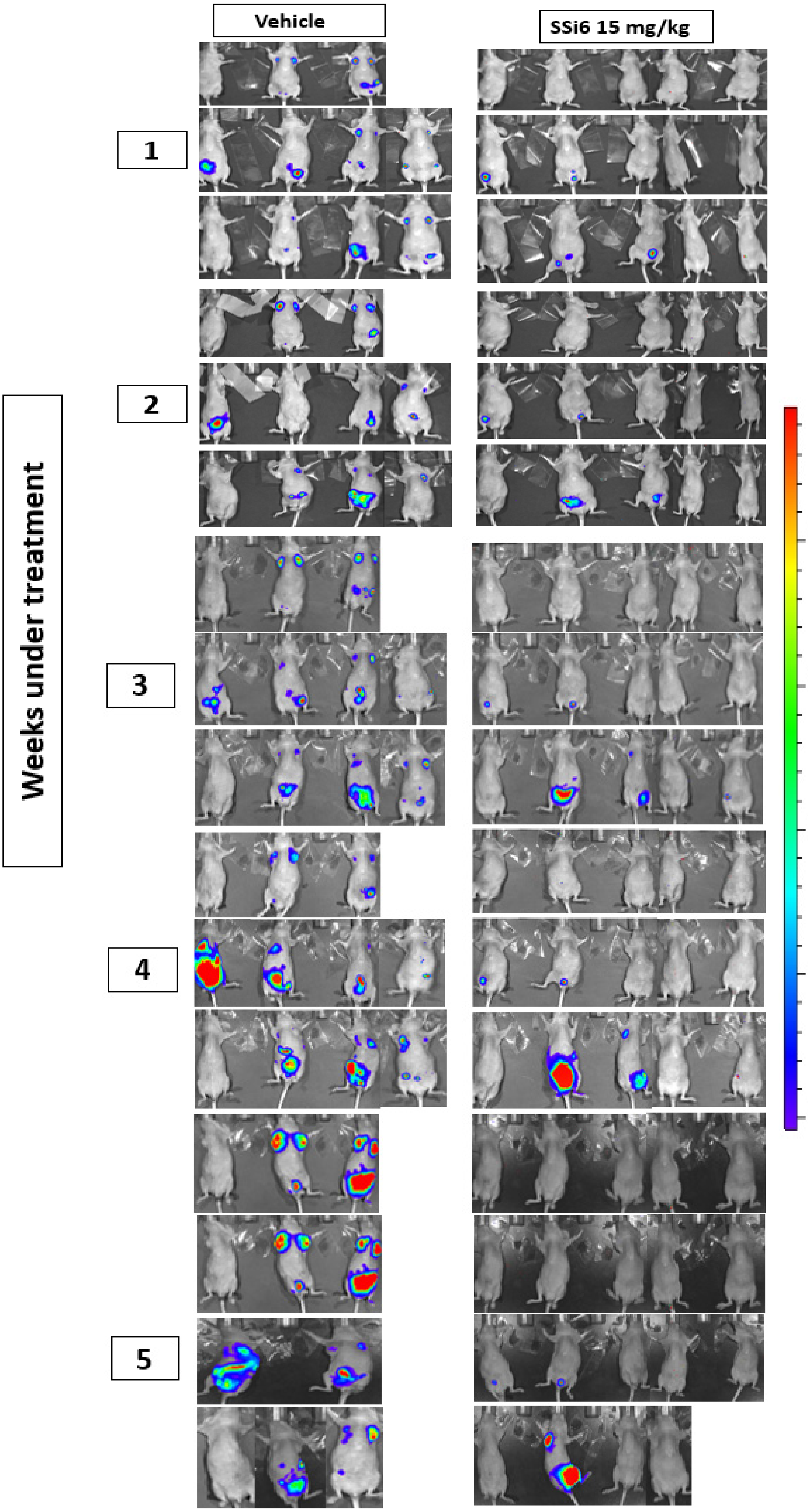

**Figure S8.** Bioluminescent images of metastasis xenograft model with resection of the primary tumor. All mice in the vehicle group (*n* = 11) and the SSi6 group (*n* = 15) were monitored for 5 weeks of treatment. The mice were injected intraperitoneally with luciferin (15 mg/mL; 200 μL) and then anesthetized with isoflurane in oxygen (2–4%), to be analyzed for metastases in the acquisition chamber of the IVIS system. (**A**) The images represent the upper segment showing the metastases in the axillary lymph nodes and thorax. The lower part was covered with a piece of black fabric to block high luminescence from the lower segment. (**B**) The lower segment showing metastasis of the abdominal area and regrowth of primary tumors. (**C**) whole-body images of the vehicle group and SSi6 (15 mg/kg).

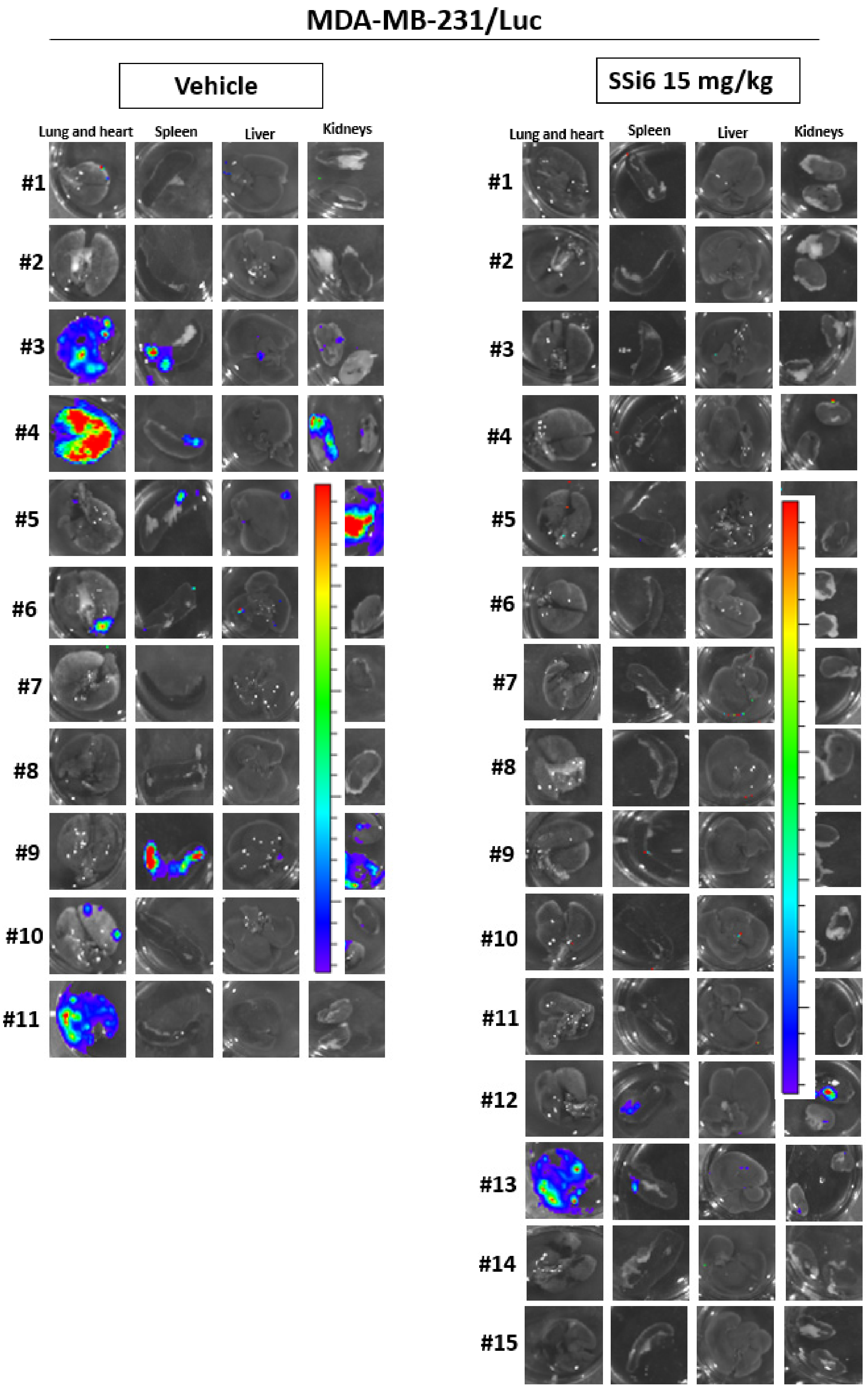
MDA-MB-231/Luc
Vehicle
SSi6 15 mg/kg
Lung and heart
Spleen
Liver
Kidneys
#1
#2
#3
#4
#5
#6
#7
#8
#9
#10
#11
#12
#13
#14
#15

**Figure S9.**SSi6 treatment prevented lung and heart, spleen, liver, kidneys metastasis in a model with primary tumor resection. Before euthanasia, the mice of vehicle and SSi6 group were injected with luciferin (see materials and methods) and the organs were analyzed by bioluminescence in the IVIS spectrum system.
